# Evaluation of Herbal Neonatal Chick Care Against Iron-induced Toxicity in Broilers

**DOI:** 10.4103/0971-6580.75858

**Published:** 2011

**Authors:** J. Chandravathy, Gopala A. Reddy, C. Haritha, B. Anilkumar

**Affiliations:** Department of Pharmacology and Toxicology, College of Veterinary Science, Rajendranagar, Hyderabad - 500 030, India

**Keywords:** Ferrous sulfate, herbal neonatal chick care, oxidative stress

## Abstract

A study was conducted to evaluate the protective effect of herbal Neonatal Chick care (NNCC) against iron-induced oxidative stress. A total of 130 day-old sexed male broiler chicks (*Vencobb* strain) were randomly divided into six groups consisting of 25 chicks each in groups 1-4 and 15 each in groups 5 and 6. Group 1 was maintained on basal diet, groups 2 and 3 on herbal NNCC at 6 and 8 g/chick/day, respectively, for 2 days immediately after hatching and later continued with basal diet up to 6 wk. Group 4 was given FeSO_4_ at 0.5% of feed for 6 wk, while groups 5 and 6 were given NNCC as in groups 2 and 3, and later continued with the FeSO_4_ as in group 4 for 6 wk. The concentration of thiobarbituric acid reactive substances (TBARS), protein carbonyls, glucose and calcium, and the activity of alanine transaminase (ALT) were significantly (*P*<0.05) increased in group 4 at the end of 6^th^ week, while the concentration of reduced glutathione (GSH), and the activities of superoxide dismutase (SOD) and catalase, phytohemagglutinin (PHA) index and HI titer were significantly (*P*<0.05) decreased in group 4. The NNCC treated groups (2, 3, 5 and 6) showed marked improvement in all the above parameters. It can be concluded that herbal NNCC offered protection and proved beneficial in resisting the adverse effects of stressor.

## INTRODUCTION

Free radicals are continuously produced by the body and are also generated through environmental pollution, radiation, drugs, chemicals, pesticides, etc.[1] Iron is the most common cofactor within the oxygen handling biological machinery[2] and interacts with molecular oxygen and generates reactive oxygen species (ROS) through Haber-Weiss and Fenton reactions,[3] leading to oxidative stress.[4] High tissue iron concentrations have been associated with the development and progression of several pathological conditions including certain cancers, liver and heart diseases, diabetes, hormonal abnormalities and immune system dysfunctions.[5] Lipid peroxidation and subsequent oxidative stress could be mitigated by antioxidants. Endogenous and passively acquired exogenous antioxidant defense systems do not accelerate in maturation until late in the third trimester.[6] Therefore, provision of proper neonatal nourishment is essential to make the organism to resist the adversities in further life. Keeping the above facts in view, an experimental study was planned to evaluate the potential of feeding herbal Neonatal Chick care (NNCC) immediately after hatch in amelioration of iron-induced oxidative stress and injury to the biological system.

## MATERIALS AND METHODS

A total of 130 day-old sexed male broiler chicks (*Vencobb* strain) were randomly divided into six groups consisting of 25 chicks each in groups 1–4 and 15 each in groups 5 and 6, to evaluate the efficacy of herbal NNCC (Ayurvet Ltd., Himachal Pradesh) in improving immune response, overall growth and development of digestive organs. All the birds were provided with feed and water *ad libitum* throughout the experiment and were maintained as per the following treatment schedule for 6 weeks:

Group 1: Basal diet control
Group 2: Herbal NNCC 6 g paste/chick/day immediately after hatch for 2 days and normal feed from 3^rd^ day onward (there was no left over paste)
Group 3: Herbal NNCC 8 g paste/chick/day immediately after hatch for 2 days and normal feed from 3^rd^ day onward (there was no left over paste)
Group 4: Basal diet + stressor (ferrous sulfate) at 0.5% in feed
Group 5: Basal diet + stressor (ferrous sulfate) + NNCC as per groups 2 and 4
Group 6: Basal diet + stressor (ferrous sulfate) + NNCC as per groups 3 and 4

Herbal NNCC is a polyherbal formulation containing *Terminalia chebula, Terminalia bellerica, Vitis vinifera, Aegle marmelos, Spirulina and Phyllanthus emblica.*

Superoxide dismutase (SOD)[7] and catalase[8] were estimated on 28^th^ and 42^nd^ days in RBC. Thiobarbituric acid reactive substances (TBARS)[9] and reduced glutathione (GSH)[10] were estimated in liver, kidney and heart, and protein carbonyls in liver,[11] and the immune status was assessed by performing HI and phytohemagglutinin (PHA) tests at the end of 6^th^ wk.

Serum samples (*n* = 8) were separated for estimation of alanine transaminase (ALT), glucose and calcium (Span Diagnostics Ltd., Surat, India) by using diagnostic kits. The data were analyzed by one-way analysis of variance (ANOVA) using statistical package for social sciences (SPSS), version 15. *P*<0.05 was considered as significant.

## RESULTS AND DISCUSSION

The TBARS concentrations [μM malondialdehyde (MDA)/mg protein] of liver, heart and kidney were significantly (*P*<0.05) increased in group 4 (0.58±0.03, 16.48±0.27, and 20.11±0.75, respectively) at the end of 6^th^ week. The TBARS concentrations of liver, heart and kidney in groups 5 and 6 showed a significant (*P*<0.05) decrease as compared to group 4 [Table 1]. The GSH concentrations (μM/mg protein) in liver, heart and kidney were significantly (*P*<0.05) decreased in group 4 (0.21±0.03, 0.17±0.01 and 2.24±0.16, respectively), while groups 5 and 6 showed a significant (*P*<0.05) increase in GSH concentration as compared to group 4 [Table 1]. The activity of erythrocytic SOD (U/mg protein) and catalase (μg H_2_ O_2_ decomposed/mg protein/min) was significantly (*P*<0.05) decreased in group 4 (10.37±0.71 and 1.29±0.18, respectively), while groups 5 and 6 showed a significant (*P*<0.05) increase in SOD and catalase activity as compared to group 4 [Table 2]. The concentration of protein carbonyls (nM/100 mg protein) in liver was significantly (*P*<0.05) increased in group 4 (19.28±0.73) at the end of 6^th^ week, while groups 5 and 6 showed a significant (*P*<0.05) decrease in protein carbonyls concentration as compared to group 4 [Table 3].

**Table 1:** Results of oxidative stress and antioxidant defenses

Group	Liver	Heart	Kidney
TBARS (µM of MDA/mg protein)			
1. Basal diet	0.12±0.02^a^	5.17±0.551^a^	8.52±0.43^a^
2. Herbal neonatal chick care	0.16±0.01^ab^	5.76±0.36^a^	9.12±0.54^a^
3. Herbal neonatal chick care	0.23±0.01^b^	5.47±0.63^a^	9.00±0.73^a^
4. FeSO_4_ + basal diet	0.58±0.03^d^	16.48±0.27^c^	20.11±0.75^c^
5. Herbal neonatal chick care + FeSO_4_ + basal diet	0.41±0.05^c^	7.98±0.47^b^	11.53±0.85^b^
6. Herbal Neonatal Chick care + FeSO_4_ + basal diet	0.45±0.06^c^	7.92±0.43^b^	13.06±0.67^b^
GSH (µM/mg protein)			
1. Basal diet	0.66±0.08^d^	0.93±0.08^d^	9.38±0.6^d^
2. Herbal neonatal chick care	0.51±0.02^c^	0.51±0.04^c^	6.12±0.59^c^
3. Herbal neonatal chick care	0.52±0.02^c^	0.47±0.04^c^	5.95±0.59^c^
4. FeSO_4_ + basal diet	0.21±0.03^a^	0.17±0.01^a^	2.24±0.16^a^
5. Herbal neonatal chick care + FeSO_4_ + basal diet	0.26±0.02^ab^	0.35±0.02^b^	3.68±0.41^ab^
6. Herbal neonatal chick care + FeSO_4_ + basal diet	0.34±0.04^b^	0.30±0.01^b^	4.63±0.61^bc^
Protein carbonyls (nM/100mg protein)			
1. Basal diet	5.68±0.84^a^	–	–
2. Herbal neonatal chick care	12.17±0.87^bc^	–	–
3. Herbal neonatal chick care	10.06±0.72^b^	–	–
4. FeSO_4_ + basal diet	19.28±0.73^d^	–	–
5. Herbal neonatal chick care + FeSO_4_ + basal diet	13.54±1.35^c^	–	–
6. Herbal neonatal chick care + FeSO_4_ + basal diet	12.05±0.91^bc^	–	–

Values are Mean±SE (*n* = 10); one-way ANOVA (SPSS). Means with different alphabets as superscripts differ significantly (*P*<0.05)

**Table 2:** Activity of SOD, catalase and ALT, and concentration of glucose and calcium

Group	4^th^ wk	6^th^ wk
SOD (U/mg protein)		
1. Basal diet	30.75±3.18^cA^	33.71±0.72^dA^
2. Herbal neonatal chick care (6 g/chick/day)	19.03±2.22^bA^	22.38±0.41^cA^
3. Herbal neonatal chick care (8 g/chick/day)	20.69±1.14^bA^	21.80±0.42^cA^
4. FeSO_4_ control	6.92±1.74^aA^	10.37±0.71^aA^
5. Herbal neonatal chick care (6 g/chick/day) + FeSO_4_	14.18±2.36^bA^	17.07±1.11^bA^
6. Herbal neonatal chick care (8 g/chick/day) + FeSO_4_	16.67±3.09^bA^	18.53±1.51^Ba^
Catalase (µg H_2_O_2_ decomposed/mg protein/min)		
1. Basal diet	8.29±0.41^eA^	9.57±0.3^dB^
2. Herbal neonatal chick care (6 g/chick/day)	6.68±0.13^dA^	7.08±0.17^cA^
3. Herbal neonatal chick care (8 g/chick/day)	5.91±0.12^cdA^	6.69±0.36^cA^
4. FeSO_4_ control	0.98±0.31^aA^	1.29±0.18^aA^
5. Herbal neonatal chick care (6 g/chick/day) + FeSO_4_	5.11±0.72^cA^	4.89±0.43^bA^
6. Herbal neonatal chick care (8 g/chick/day) + FeSO_4_	3.98±0.25^bA^	5.08±0.27^bB^
ALT (U/l)		
1. Basal diet	6.98±3.01^aA^	8.08±7.12^aA^
2. Herbal neonatal chick care (6 g/chick/day)	9.89±1.47^aA^	11.35±6.78^aA^
3. Herbal neonatal chick care (8 g/chick/day)	11.20±1.91^aA^	14.56±5.23^aA^
4. FeSO_4_ control	56.38±5.40^CA^	69.67±2.34^cA^
5. Herbal neonatal chick care (6 g/chick/day) + FeSO_4_	36.12±6.72^bA^	44.34±7.64^bA^
6. Herbal neonatal chick care (8 g/chick/day) + FeSO_4_	34.14±3.56^bA^	36.37±9.00^bA^
Glucose (mg/dl)		
1. Basal diet	35.34±1.28^aA^	36.75±1.78^aA^
2. Herbal neonatal chick care (6 g/chick/day)	41.30±1.47^abA^	41.68±1.82^abA^
3. Herbal neonatal chick care (8 g/chick/day)	45.00±1.56^bA^	52.80±2.06^cA^
4. FeSO_4_ control	65.88±3.8^dA^	65.58±2.83^dA^
5. Herbal neonatal chick care (6 g/chick/day) + FeSO_4_	45.69±2.27^bA^	53.40±4.06^cA^
6. Herbal neonatal chick care (8 g/chick/day) + FeSO_4_	52.22±1.89^cA^	54.85±3.27^bcA^
Calcium (mg/dl)		
1. Basal diet	2.55±0.27^aA^	6.01±0.78^aB^
2. Herbal neonatal chick care (6 g/chick/day)	2.96±0.23^aA^	7.01±0.44^aB^
3. Herbal neonatal chick care (8 g/chick/day)	3.59±0.17^aA^	6.93±0.51^aB^
4. FeSO_4_ control	3.93±0.52^bA^	9.06±0.31^aB^
5. Herbal neonatal chick care (6 g/chick/day) + FeSO_4_	3.96±0.44^bA^	9.43±0.16^aB^
6. Herbal neonatal chick care (8 g/chick/day) + FeSO_4_	4.79±0.41^bA^	8.93±0.24^aB^

Values are Mean±SE (*n* = 8); one-way ANOVA (SPSS). Means with different alphabets as superscripts differ significantly (*P*<0.05). Capital alphabets (horizontal comparison); small alphabets (vertical comparison); SOP, superoxide dismutase; ALT, alanine transaminase

**Table 3:** Results of immunological parameters

Group	6^th^ wk
HI (Log^2^) titer	
1. Basal diet	5.67±0.33^c^
2. Herbal neonatal hick care (6 g/chick/day)	4.67±0.61^c^
3. Herbal neonatal chick care (8 g/chick/day)	4.86±0.34^c^
4. FeSO_4_ control	2.50±0.22^a^
5. Herbal neonatal chick care (6 g/chick/day) + FeSO_4_	4.17±0.60^b^
6. Herbal neonatal chick care (8 g/chick/day) + FeSO_4_	4.23±0.75^b^
Phytohemagglutinin (PHA) assay (mm)	
1. Basal diet	0.85±0.04^d^
2. Herbal neonatal chick care (6 g/chick/day)	0.68±0.03^c^
3. Herbal neonatal chick care (8 g/chick/day)	0.73±0.06^c^
4. FeSO_4_ control	0.22±0.06^a^
5. Herbal neonatal chick care (6 g/chick/day) + FeSO_4_	0.52±0.03^b^
6. Herbal neonatal chick care (8 g/chick/day) + FeSO_4_	0.48±0.03^b^

Values are Mean±SE (*n* = 8); one-way ANOVA (SPSS). Means with different alphabets as superscripts differ significantly (*P*<0.05)

The TBARS and protein carbonyls were elevated and the antioxidant defenses like GSH, SOD and catalase were decreased in the toxic control group 4 that was maintained on iron throughout the study. All the changes in the antioxidant defense profile were significantly reversed in the groups (5 and 6) that were treated with herbal NNCC. These results may be attributed to the antioxidant properties aqueous extract of *T. chebula>*,[12] *T. bellerica*,[13] grape seed proanthocyanidines,[14] *A. marmelos*,[15] *Spirulina plantensis*,[16] etc. Therefore, the polyherbal formulation, NNCC, showed potent antioxidant actions in this study.

The activity of ALT (U/l) and the concentration of glucose (mg/dl) was significantly (*P*<0.05) increased on 4^th^ and 6^th^ weeks in group 4 as compared to group 1 [Table 2], while groups 5 and 6 revealed significant (*P*<0.05) decrease as compared to group 4. The increased activity of ALT and concentration of glucose in the iron toxic control group 4 suggests the hapatocellular insult following administration of iron. Treatment with herbal NNCC resulted in significant reduction in the activity of ALT. The hepatocellular injury due to iron could be attributed to the iron-induced generation of ROS or free radicals and the reversal of the findings following treatment could be attributed to the antioxidant and the hepatoprotective potential of the ingredients of herbal NNCC, namely, *A. marmelos*,[17] *T. chebula*,[18] *P. emblica*,[19] etc.

The PHA index (mm) and HI titer (Log^2^) were significantly (*P*<0.05) decreased in the iron control group 4 (0.22±0.06 and 2.50±0.22, respectively) [Table 3], while the thickness and HI titer in groups 2, 3, 5 and 6 were significantly (*P*<0.05) increased as compared to group 4. The beneficial immune competent actions of NNCC may be attributed to its synergistic herbs.
